# Supplementary material for: Global census of the significance of giant mesopelagic protists to the marine carbon and silicon cycles
Source: Nat Commun. 2024 Apr 29;15:3341. doi: 10.1038/s41467-024-47651-4 (PMC11058905; doi:10.1038/s41467-024-47651-4)
Supplement: Supplementary file 1 — Supplementary Information [file 41467_2024_47651_MOESM1_ESM.pdf]

Global census of the significance of giant mesopelagic protists to the marine carbon and silicon cycles

Supplementary Information

Manon Laget[1*], Laetitia Drago[2], Thelma Panaïotis[2], Rainer Kiko[3], Lars Stemmann[2], Andreas Rogge[4], Natalia Llopis-Monferrer[5], Aude Leynaert[6], Jean-Olivier Irisson[2], Tristan Biard[1]

[1] LOG, Laboratoire d'Océanologie et de Géosciences, Univ. Littoral Côte d'Opale, Univ. Lille, CNRS, IRD, UMR 8187, Wimereux, France
[2] Sorbonne Université, CNRS, Laboratoire d'Océanographie de Villefranche (LOV), F-06230 Villefranche-sur-Mer, France
[3] GEOMAR Helmholtz Center for Ocean Research Kiel, Kiel, Germany
[4] Section Benthic Ecology, Alfred Wegener Institute Helmholtz Center for Polar and Marine Research (AWI), Bremerhaven, Germany
[5] Monterey Bay Aquarium Research Institute, Moss Landing, CA, USA
[6] Université de Brest, CNRS, IRD, Ifremer, LEMAR, Plouzané, France
[*] Corresponding author

Corresponding author email: manon.laget@protonmail.com

Supplementary Table 1: Cruise information, number of Underwater Vision Profiler 5 profiles in each layer and presence/absence in previous studies ([1]; [2]).

Sampling cruise name	Year	Chief scientists	Regions	Profiles 0 – 200 m	Profiles 200 – 1000 m	Present in [1]	Present in [2]
BOUM	2008	T. Moutin	Mediterranean Sea	159	29	Yes	Yes
CCELTER		M. Landry	California Upwelling	74	11	Yes	Yes
OPEREX		Z. Kolber	Northern Pacific	73	7	–	Yes
MALI	2009	M. Babin	Arctic	106	12	Yes	Yes
Tara Oceans		Tara Ocean Cons.	Mediterranean Sea	35	12	Yes	Yes
LOHAFEX		V. Smetacek	Southern Atlantic	56	24	Yes	Yes
Tara Oceans	2010	Tara Ocean Cons.	Indian Ocean, Southern Atlantic	158	51	Yes	Yes
CCELTER	2011	M. Landry	California Upwelling	56	2	–	Yes
Tara Oceans		Tara Ocean Cons.	Pacific Ocean	218	58	Yes	Yes
KEOPS II		B. Queguiner	Southern Ocean	12	–	Yes	Yes
CCELTER	2012	M. Landry	California Upwelling	60	2	–	Yes
Tara Oceans		Tara Ocean Cons.	Northern Atlantic	103	34	Yes	Yes
MSM22		P. Brandt	Equatorial Atlantic	106	80	Yes	Yes
MSM23		M. Visbeck	.	64	64	Yes	Yes
AN1304	2013	M-H. Forget	Arctic	12	–	–	Yes
Tara Oceans		Tara Ocean Cons.	.	116	21	Yes	Yes
DEWEX		P. Testor, P. Conan	Mediterranean Sea	13	12	–	Yes
MOOSE-GE		MOOSE Network	.	4	–	–	Yes
M92		S. Sommer	Peru Upwelling	16	5	–	Yes
M93		G. Lavik	.	84	31	–	Yes
M96		J. Karstensen	Equatorial Atlantic	77	56	Yes	Yes
M97		T. Tanhua	.	168	110	–	–
AN1405	2014	M-H. Forget	Arctic	27	1	–	Yes
MSM40		J. Karstensen	.	5	5	–	Yes
CCELTER		M. Landry	California Upwelling	57	7	–	Yes
M107		S. Sommer	Canary Upwelling	45	12	–	Yes
MOOSE-GE		MOOSE Network	Mediterranean Sea	4	4	–	Yes
SOMBA		L. Mortier, N. Ait Ameur, V. Taillandier	.	7	6	–	Yes
M108		R. Lampitt	Northern Atlantic	11	3	–	Yes
SARGASSO		P. Munk	.	82	–	–	Yes
M105		T. Tanhua	Equatorial Atlantic	6	4	–	Yes
PS88b		J. Hahn	.	39	39	–	Yes
M106		P. Brandt	Southern Atlantic	107	98	–	Yes
GREENEDGE IceCamp	2015	M. Babin	Arctic	29	–	–	Yes
M120		M. Dengler	Benguela Upwelling	1	–	–	–
M121		M. Frank	.	60	29	–	Yes
DY032		F. Carlotti	Mediterranean Sea	15	14	–	Yes
MOOSE-GE		MOOSE Network	.	5	4	–	Yes
CASSIOPEE		F. Marin, S. Cravatte	Eastern Pacific	13	11	–	Yes
OUTPACE		T. Moutin, S. Bonnet	.	188	18	–	Yes
P16N		J. Cross	Pacific Ocean	186	177	–	Yes
M116		M. Visbeck	Equatorial Atlantic	82	81	–	Yes
M119		P. Brandt	.	49	47	–	–
GREENEDGE	2016	M. Babin	Arctic	161	43	–	Yes
PS99		A. Rogge, A. Waite	.	19	15	–	–
CCELTER		M. Landry	California Upwelling	52	3	–	Yes
M131		P. Brandt	Benguela Upwelling	18	5	–	–
AMES II		L. Karp-Boss, E. Boss	Northern Atlantic	42	14	–	Yes
M130		M. Dengler	Equatorial Atlantic	111	95	–	–
CCELTER	2017	M. Ohman	California Upwelling	72	3	–	Yes
FLUXES I		J. Aristegui	Canary Upwelling	66	37	–	Yes
FLUXES II		J. Aristegui	.	49	47	–	–
M135		T. Tanhua	Peru Upwelling	133	108	–	Yes

M138		H. Bange	.	32	–	–	Yes
MSM60		A. Rogge, A. Waite	Southern Atlantic	123	118	–	Yes
ARCTIC	2018	A. Rogge	Arctic	107	58	–	–
IPS		M. Babin	.	7	4	–	Yes
EXPORTS		L. Karp-Boss, E. Boss	Northern Pacific	75	35	–	–
EXPORTS		A. McDonnell	.	107	105	–	–
SR1812		A. Macdonald	Southern Ocean	131	12	–	–
CCELTER	2019	M. Ohman	California Upwelling	31	28	–	Yes
CurtinUni IIOE		D. Antoine	Indian Ocean	51	26	–	–
PS124	2021	A. Rogge, M. Holtappels	Southern Ocean	77	22	–	–
**Total**				**4,252**	**1,959**		

Supplementary Table 2: Characteristics of Rhizaria taxa considered in this study. ESD stands for Equivalent Spherical Diameter.

**Group**	**Order**	**Taxon**	**Test nature**	**Count epi.**	**Count meso.**	**Median ESD (μm)**
Phaeodaria		Aulacanthidae	silicified	1,875	43,897	1,479
		Aulosphaeridae	silicified	29,818	20,910	1,799
		Cannosphaeridae	silicified	441	1,889	1,568
		Castanellidae	silicified	2,398	1,061	1,357
		Circoporidae	silicified	1	585	2,009
		Coelodendridae	silicified	344	6,698	2,010
		Medusettidae	silicified	79	563	2,880
		Phaeodaria_unknown	silicified	6,049	4,116	1,355
		Tuscaroridae	silicified	2	107	5,586
Radiolaria	Acantharia	Acantharea	strontium	15,167	1,956	1,276
		Acantharea_like	strontium	659	228	1,364
	Collodaria	Collodaria_colonial	naked	3,111	219	3,108
		Collodaria_solitaryblack	naked	6,105	1,804	1,880
		Collodaria_solitaryglobule	naked	2,532	403	2,521
	Orodaria	Orodaria_other	silicified	1	43	3,535
		Cytocladus	silicified	2	14	7,097
Foraminifera		Foraminifera	calcite	1,474	3,244	2,052
Rhizaria_other		Rhizaria_like	unknown	4,099	5,657	1,481
			**Total**	74,157	93,394	

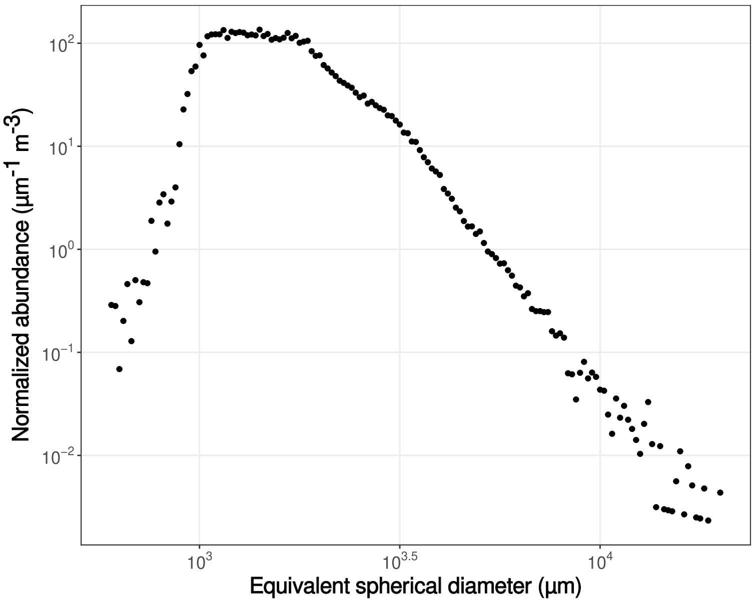

Supplementary Figure 1: Normalized abundance size spectrum including all planktonic Rhizaria specimens considered in this study.

Supplementary Table 3: Results of the carbon models for each Rhizaria taxon as well as for all large Rhizaria together. CV stands for cross-validation. No model is available for the order Orodaria in the epipelagic layer as no specimen was sampled there.

Layer	Taxon	R^2 (random CV)	R^2 (spatial CV)	Mean integrated biomass (mg C m^{-2})	Total biomass (Tg C)
Epipelagic	Acantharia	15.3	5.0	1.11 ± 1.90	0.48 (0.42-0.55)
	Collodaria	34.6	6.1	3.52 ± 3.31	1.50 (1.32-1.69)
	Foraminifera	47.8	1.5	0.50 ± 0.83	0.23 (0.19-0.27)
	Orodaria	–	–	–	–
	Phaeodaria	36.9	0.1	2.15 ± 6.61	0.66 (0.57-0.76)
	Rhizaria_other	52.6	0.2	0.50 ± 0.91	0.20 (0.17-0.23)
Mesopelagic	Acantharia	42.9	<0.1	0.29 ± 0.49	0.10 (0.08-0.12)
	Collodaria	30.5	<0.1	1.28 ± 1.41	0.46 (0.37-0.54)
	Foraminifera	30.4	1.6	0.80 ± 1.15	0.34 (0.28-0.40)
	Orodaria	0.9	0.0	0.02 ± 0.17	0.01 (0.00-0.01)
	Phaeodaria	33.3	7.4	21.61 ± 22.67	7.28 (6.53-8.03)
	Rhizaria_other	36.7	9.4	1.34 ± 2.04	0.42 (0.36-0.47)
Epipelagic	All Rhizaria	47.3	0.1	8.84 ± 10.16	3.50 (3.16-3.84)
Mesopelagic	All Rhizaria	48.6	5.4	26.31 ± 25.98	8.93 (8.06-9.80)

Supplementary Table 4: Results of the models for biogenic silica (bSi) biomass and production as well as carbon (C) demand for all Phaeodaria. CV stands for cross-validation.

Layer	Measure	R^2 (random CV)	R^2 (spatial CV)	Total value
Epipelagic	bSi biomass	33.3	0.12	0.33 Tg Si
	bSi production	34.0	0.02	0.70 Tg Si y^{-1}
Mesopelagic	C demand	38.2	10.7	0.46 Pg C y^{-1}
	bSi biomass	32.3	9.5	3.91 Tg Si
	bSi production	40.6	7.4	3.96 Tg Si y^{-1}

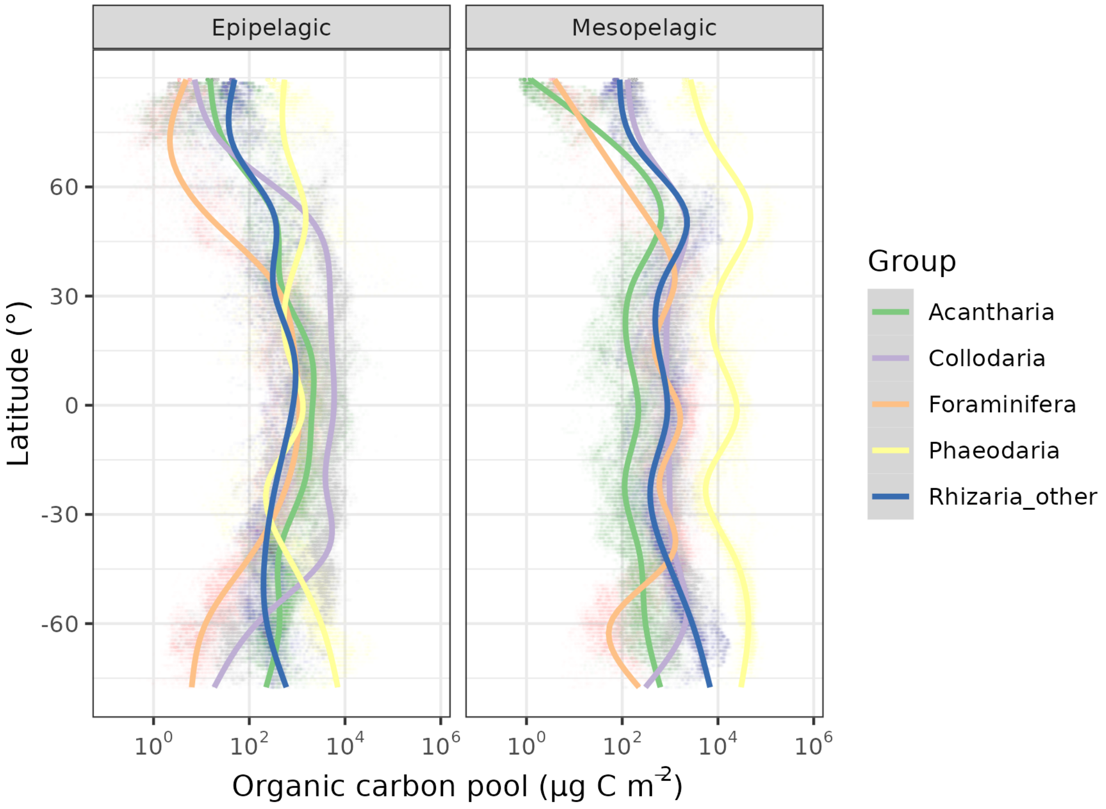

Supplementary Figure 2: Integrated carbon biomass as a function of latitude for the epipelagic and mesopelagic layers for main Rhizaria groups. Regression curves were derived using Generalized Additive Models. Only groups whose model's R^2 calculated by random cross-validation is >0.05 are shown.

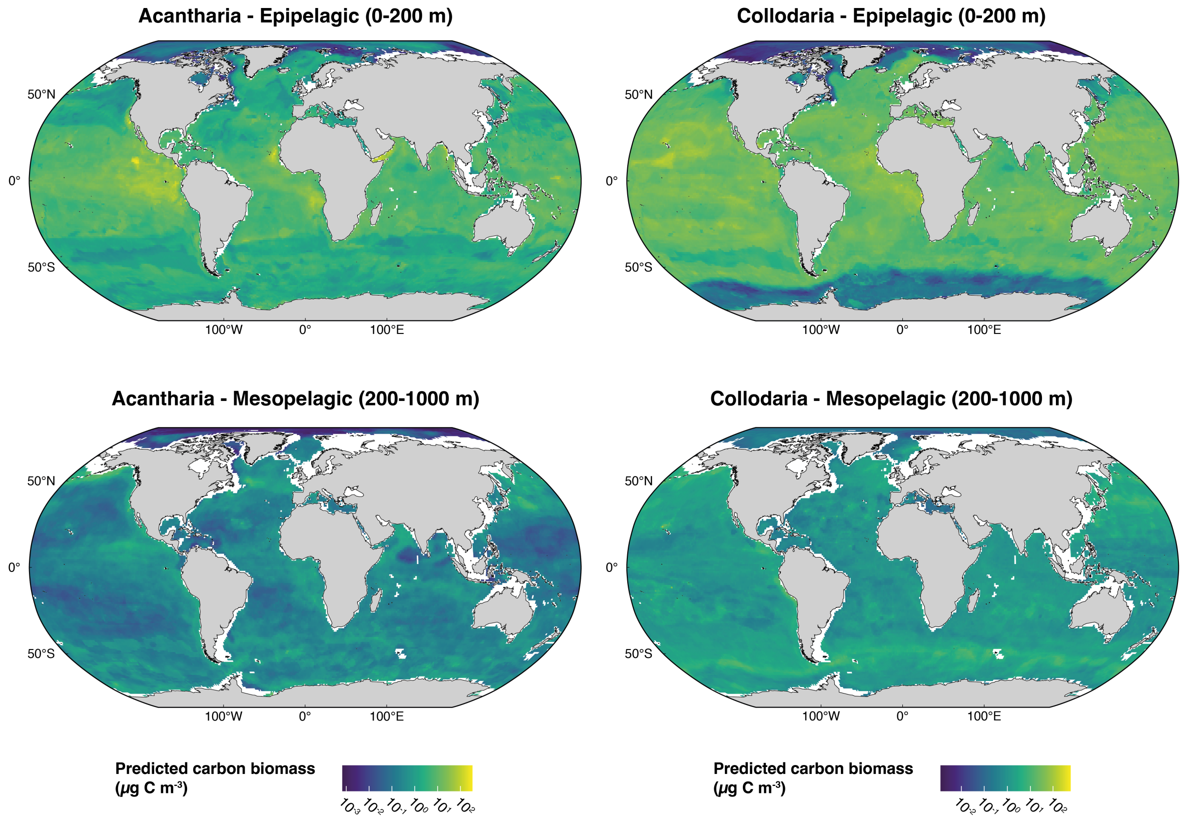

Supplementary Figure 3: Maps of the predicted average 1° × 1° carbon concentration in the epipelagic layer (0-200 m) and in the mesopelagic layer (200-1,000 m) for Acantharia and Collodaria. All maps were created using the R software version 4.0.3 [3].

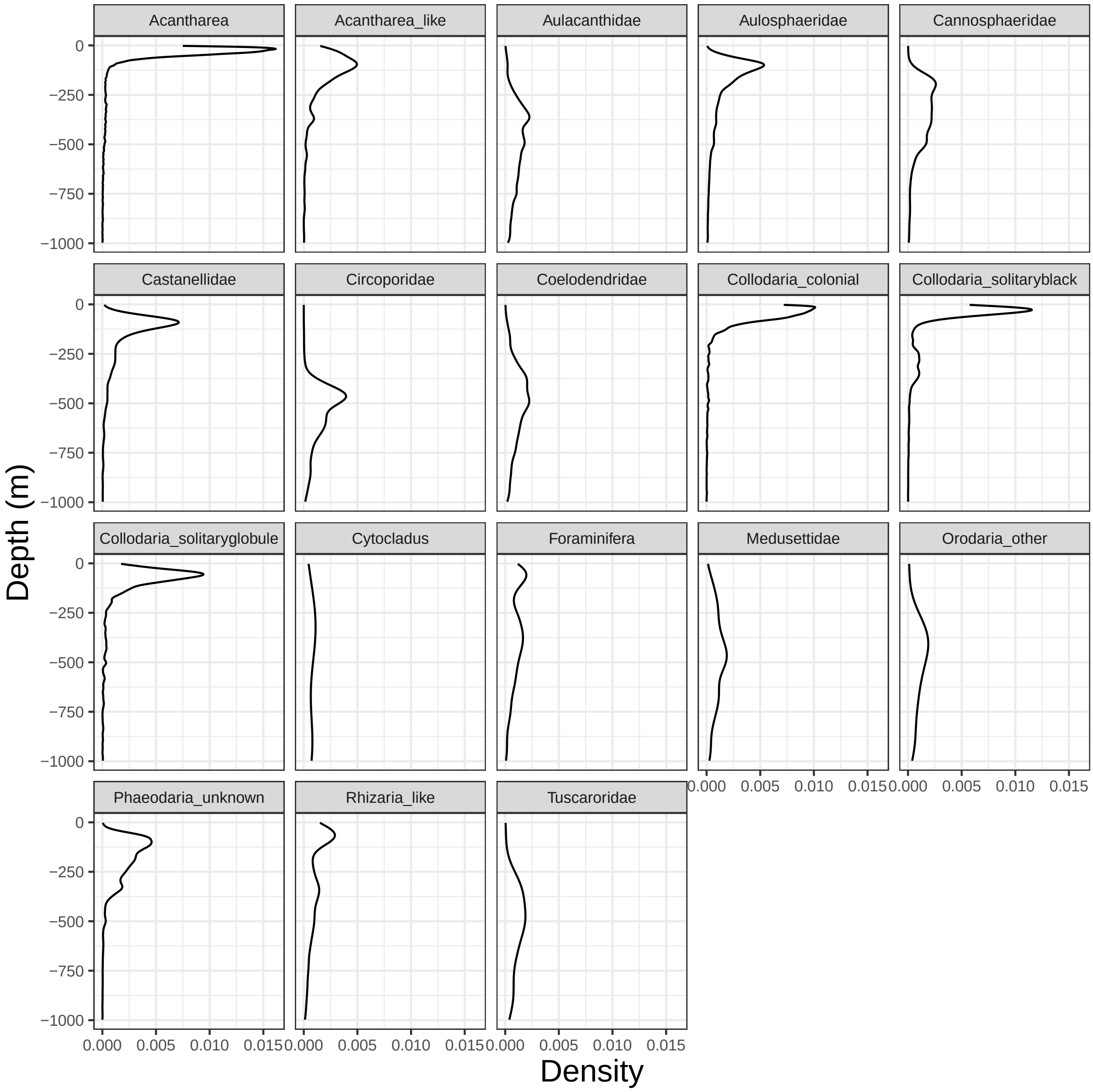

Supplementary Figure 4: Distribution of sampled Rhizaria specimens according to depth, for each taxon considered.

Supplementary Table 5: Global and regional carbon demand of large mesopelagic Phaeodaria. Numbers between brackets refer to references listed in the Supplementary References at the end of this document.

Area	Total C demand (Pg C y^{-1})	Mean integrated demand (mg C m^{-2} d^{-1})	Carbon export (Pg C y^{-1}) and associated references
World	0.46	3.9 ± 3.4	5 [4] - 12 [5]
Arctic	0.003	1.1 ± 0.6	0.15 [5] - 0.51 [6]
Subarctic Atlantic	0.006	2.0 ± 1.2	NA
Subarctic Pacific	0.037	13.0 ± 6.5	NA
North Atlantic	0.026	2.9 ± 2.5	1.52 [6]
North Pacific	0.055	4.0 ± 4.7	1.74 [6]
Tropical and upwelling Atlantic	0.030	6.1 ± 3.4	0.83 [6]
Tropical and upwelling Pacific	0.073	4.1 ± 3.3	1.09 [6]
South Atlantic	0.015	2.2 ± 1.7	0.75 [6]
South Pacific	0.035	2.4 ± 2.1	0.61 [6]
Indian Ocean	0.040	2.6 ± 1.4	1.54 [6]
Southern Ocean	0.145	5.2 ± 1.9	0.62 [5] - 1.3 [5]

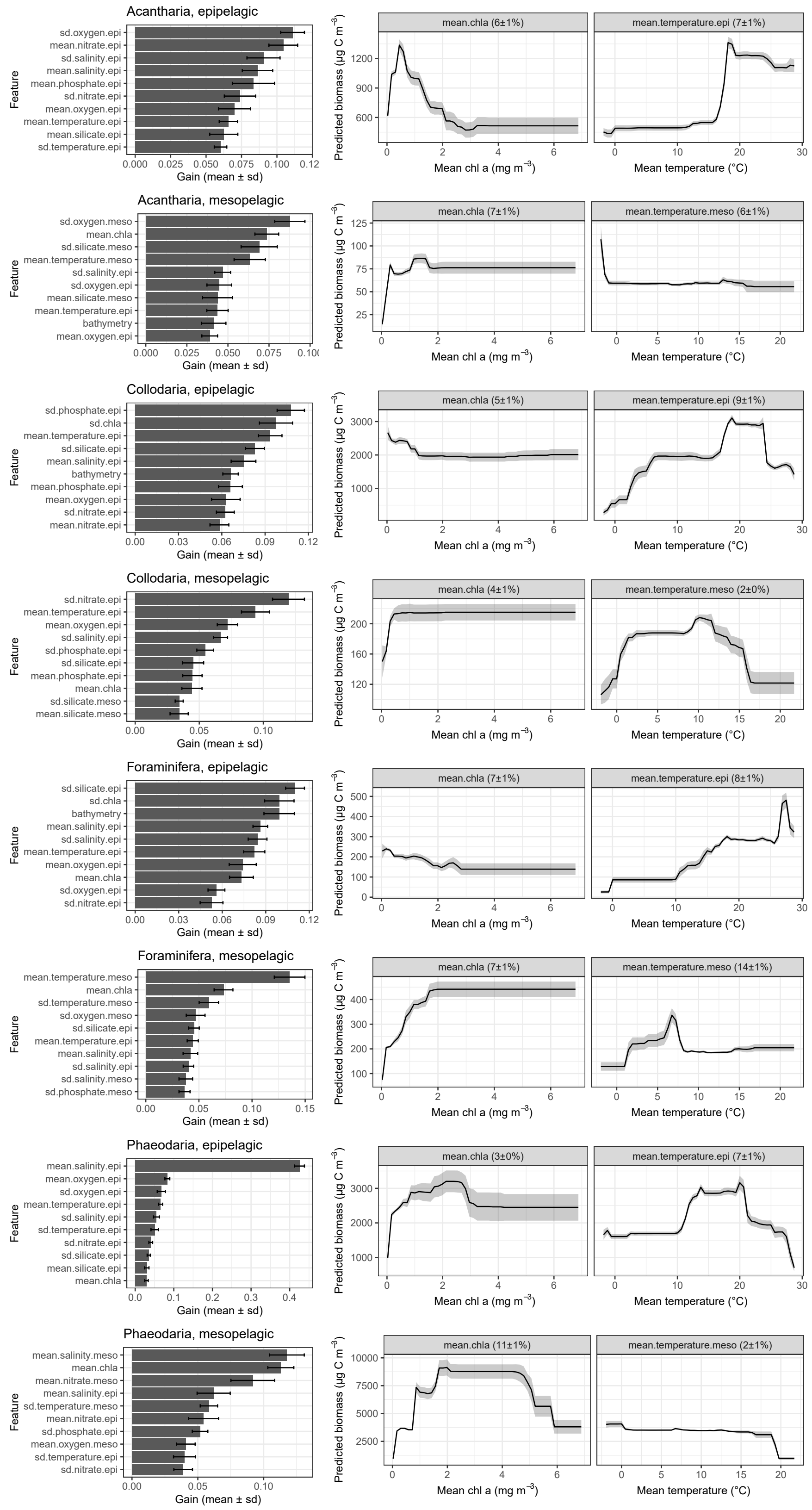

Supplementary Figure 5: Order of importance of the 10 most important variables in the models for the epipelagic (0-200 m) and mesopelagic (200-1000 m) layers for main Rhizaria groups (left). Partial dependence plots of the mean chlorophyll a concentration and mean temperature are shown on the right.

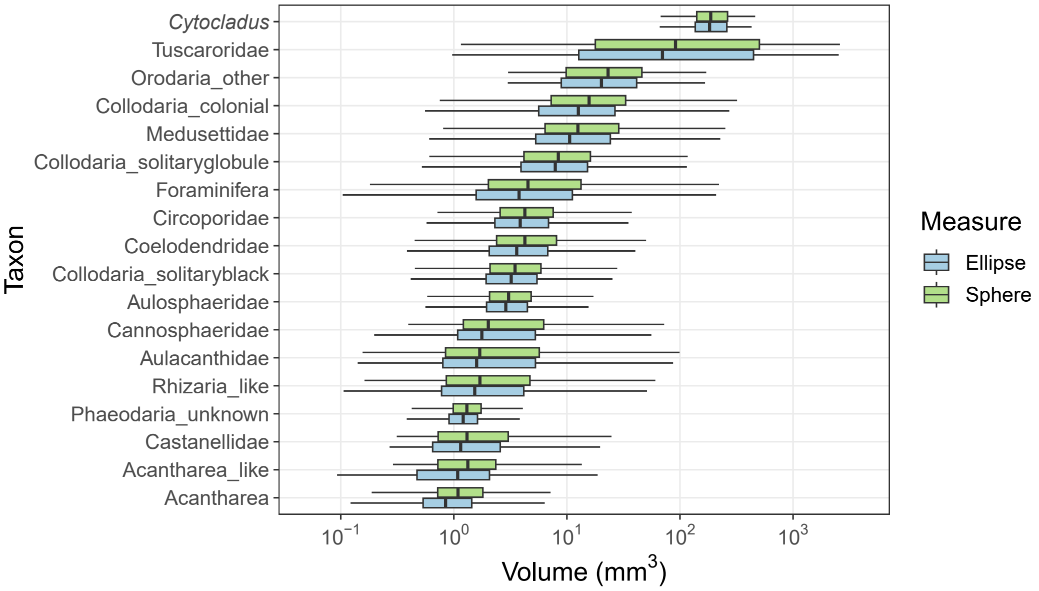

Supplementary Figure 6: Volume distributions for each Rhizaria taxa considered when using the ellipse (blue) or sphere (green) method. Boxes' bottom and top boundaries represent the 25th and 75th percentiles. Lower whiskers stretch from the 25th percentile to the minimum value within 1.5 times the 25th percentile's inter-quartile range (IQR). Upper whiskers extend from the 75th percentile to the maximum value within 1.5 times the 75th percentile's IQR.

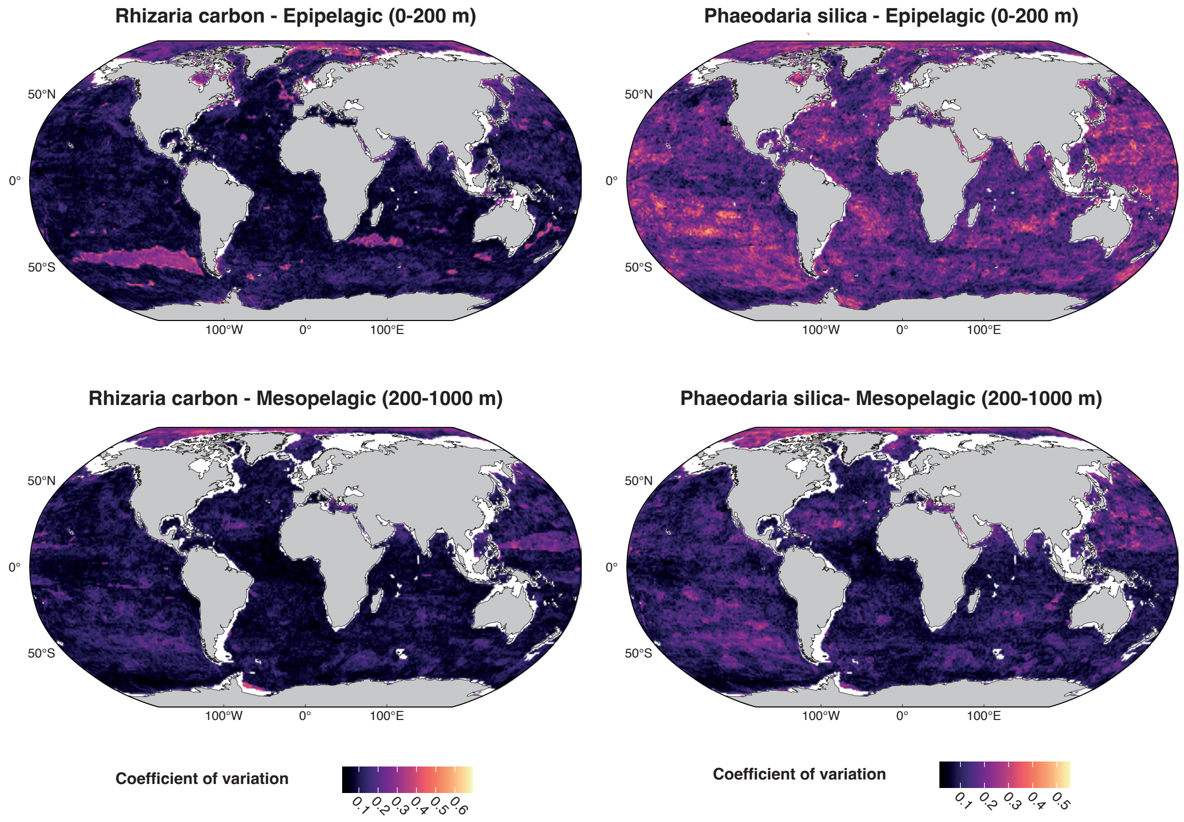

Supplementary Figure 7: Maps of the coefficient of variation of the predicted 1° × 1° carbon concentration in the epipelagic layer (0-200 m) and in the mesopelagic layer (200-1,000 m) for all Rhizaria, and of the coefficient of variation of the predicted 1° × 1° silica concentration in the epipelagic layer (0-200 m) and in the mesopelagic layer (200-1,000 m) for Phaeodaria. All maps were created using the R software version 4.0.3 [3].

Supplementary References

1. Biard, T. *et al.* In situ imaging reveals the biomass of giant protists in the global ocean. *Nature* **532,** 504–507 (2016).
2. Drago, L. *et al.* Global Distribution of Zooplankton Biomass Estimated by In Situ Imaging and Machine Learning. *Frontiers in Marine Science* **9** (2022).
3. R Core Team. R: a language and environment for statistical computing. *Foundation for Statistical Computing* (2020).
4. Henson, S. A. *et al.* A reduced estimate of the strength of the ocean's biological carbon pump. *Geophysical Research Letters* **38** (2011).
5. Laws, E. A., Falkowski, P. G., Smith Jr, W. O., Ducklow, H. & McCarthy, J. J. Temperature effects on export production in the open ocean. *Global biogeochemical cycles* **14,** 1231–1246 (2000).
6. Dunne, J. P., Sarmiento, J. L. & Gnanadesikan, A. A synthesis of global particle export from the surface ocean and cycling through the ocean interior and on the seafloor. *Global Biogeochemical Cycles* **21** (2007).
