# Supplementary material for: Global census of the significance of giant mesopelagic protists to the marine carbon and silicon cycles
Source: Nat Commun. 2024 Apr 29;15:3341. doi: 10.1038/s41467-024-47651-4 (PMC11058905; doi:10.1038/s41467-024-47651-4)
Supplement: Supplementary file 3 — Reporting Summary [file 41467_2024_47651_MOESM3_ESM.pdf]

nature portfolio

Corresponding author(s): Manon Laget

Last updated by author(s): Mar 13, 2024

Reporting Summary

Nature Portfolio wishes to improve the reproducibility of the work that we publish. This form provides structure for consistency and transparency in reporting. For further information on Nature Portfolio policies, see our Editorial Policies and the Editorial Policy Checklist.

Statistics

For all statistical analyses, confirm that the following items are present in the figure legend, table legend, main text, or Methods section.

n/a	Confirmed	
☐	☒	The exact sample size (*n*) for each experimental group/condition, given as a discrete number and unit of measurement
☐	☒	A statement on whether measurements were taken from distinct samples or whether the same sample was measured repeatedly
☒	☐	The statistical test(s) used AND whether they are one- or two-sided<br>*Only common tests should be described solely by name; describe more complex techniques in the Methods section.*
☒	☐	A description of all covariates tested
☒	☐	A description of any assumptions or corrections, such as tests of normality and adjustment for multiple comparisons
☐	☒	A full description of the statistical parameters including central tendency (e.g. means) or other basic estimates (e.g. regression coefficient) AND variation (e.g. standard deviation) or associated estimates of uncertainty (e.g. confidence intervals)
☒	☐	For null hypothesis testing, the test statistic (e.g. *F*, *t*, *r*) with confidence intervals, effect sizes, degrees of freedom and *P* value noted<br>*Give P values as exact values whenever suitable.*
☒	☐	For Bayesian analysis, information on the choice of priors and Markov chain Monte Carlo settings
☒	☐	For hierarchical and complex designs, identification of the appropriate level for tests and full reporting of outcomes
☒	☐	Estimates of effect sizes (e.g. Cohen's *d*, Pearson's *r*), indicating how they were calculated

Our web collection on statistics for biologists contains articles on many of the points above.

Software and code

Policy information about availability of computer code

Data collection	Rhizaria images were validated by taxonomy experts on the EcoTaxa web application.
Data analysis	We performed analyses using the R software version 4.0.3, the xgboost package version 1.2.0.1 for boosted regression trees, and the blockCV package for cross-validation. Analyses were performed using custom scripts available in the Zenodo repository https://doi.org/10.5281/zenodo.10652050.

For manuscripts utilizing custom algorithms or software that are central to the research but not yet described in published literature, software must be made available to editors and reviewers. We strongly encourage code deposition in a community repository (e.g. GitHub). See the Nature Portfolio guidelines for submitting code & software for further information.

Data

Policy information about availability of data

All manuscripts must include a data availability statement. This statement should provide the following information, where applicable:

- Accession codes, unique identifiers, or web links for publicly available datasets
- A description of any restrictions on data availability
- For clinical datasets or third party data, please ensure that the statement adheres to our policy

The input and output model data generated in this study have been deposited in the Zenodo database [https://doi.org/10.5281/zenodo.10652050]. The environmental data used in this study are available in the World Ocean Atlas database at https://www.ncei.noaa.gov/products/world-ocean-atlas. The surface chlorophyll a data are available in the Copernicus database at https://data.marine.copernicus.eu/product/OCEANCOLOUR_GLO_BGC_L4_NRT_009_102.

Research involving human participants, their data, or biological material

Policy information about studies with human participants or human data. See also policy information about sex, gender (identity/presentation), and sexual orientation and race, ethnicity and racism.

Reporting on sex and gender	N/A
Reporting on race, ethnicity, or other socially relevant groupings	N/A
Population characteristics	N/A
Recruitment	N/A
Ethics oversight	N/A

Note that full information on the approval of the study protocol must also be provided in the manuscript.

Field-specific reporting

Please select the one below that is the best fit for your research. If you are not sure, read the appropriate sections before making your selection.

☐ Life sciences ☐ Behavioural & social sciences ☒ Ecological, evolutionary & environmental sciences

For a reference copy of the document with all sections, see nature.com/documents/nr-reporting-summary-flat.pdf

Ecological, evolutionary & environmental sciences study design

All studies must disclose on these points even when the disclosure is negative.

Study description	We used a global Underwater Vision Profiler 5 (UVP5) dataset, comprising 4,252 vertical profiles and 167,551 validated Rhizaria images collected throughout the world ocean. Boosted regression trees were applied to model on a global scale the organic carbon and biogenic silica biomass, as well as the carbon demand and biogenic silica production of these organisms in both the epipelagic and mesopelagic layers. We compared carbon demand estimates to literature values of carbon export to estimate carbon flux attenuation mediated by these organisms in the mesopelagic layer.
Research sample	The dataset is composed of 4,252 UVP5 vertical profiles which include 167,551 human-validated Rhizaria images collected throughout the world ocean. This is a compilation of most UVP5 data, collected during 64 oceanographic cruises occurring between 2008 and 2021. This dataset allows an extensive coverage of the world ocean. To estimate carbon flux attenuation, we compiled all available regional and global carbon export values from the literature, to get a representative quantification of these values.
Sampling strategy	The UVP5 was routinely deployed during the involved cruises, to get an extensive dataset of vertical profiles.
Data collection	Data were collected by performing vertical profiles of the Underwater Vision Profiler 5 during various cruises which took place in all oceans between 2008 and 2021.
Timing and spatial scale	Data were collected between 2008 and 2021. As no regularity could be achieved given the nature of the dataset, they were averaged over a 1°x1° global grid over all years.
Data exclusions	No piece of data was excluded from analyses.
Reproducibility	Data and codes to reproduce the results are made publicly available.
Randomization	Not relevant as no comparison between groups is involved.

Blinding	Not relevant as no comparison between groups is involved.

Did the study involve field work? ☐ Yes ☒ No

Reporting for specific materials, systems and methods

We require information from authors about some types of materials, experimental systems and methods used in many studies. Here, indicate whether each material, system or method listed is relevant to your study. If you are not sure if a list item applies to your research, read the appropriate section before selecting a response.

Materials & experimental systems

n/a	Involved in the study
☒	☐ Antibodies
☒	☐ Eukaryotic cell lines
☒	☐ Palaeontology and archaeology
☒	☐ Animals and other organisms
☒	☐ Clinical data
☒	☐ Dual use research of concern
☒	☐ Plants

Methods

n/a	Involved in the study
☒	☐ ChIP-seq
☒	☐ Flow cytometry
☒	☐ MRI-based neuroimaging

Plants

Seed stocks	*Report on the source of all seed stocks or other plant material used. If applicable, state the seed stock centre and catalogue number. If plant specimens were collected from the field, describe the collection location, date and sampling procedures.*
Novel plant genotypes	*Describe the methods by which all novel plant genotypes were produced. This includes those generated by transgenic approaches, gene editing, chemical/radiation-based mutagenesis and hybridization. For transgenic lines, describe the transformation method, the number of independent lines analyzed and the generation upon which experiments were performed. For gene-edited lines, describe the editor used, the endogenous sequence targeted for editing, the targeting guide RNA sequence (if applicable) and how the editor was applied.*
Authentication	*Describe any authentication procedures for each seed stock used or novel genotype generated. Describe any experiments used to assess the effect of a mutation and, where applicable, how potential secondary effects (e.g. second site T-DNA insertions, mosiacism, off-target gene editing) were examined.*
